# Rapid Molecular Testing for TB to Guide Respiratory Isolation in the U.S.: A Cost-Benefit Analysis

**DOI:** 10.1371/journal.pone.0079669

**Published:** 2013-11-20

**Authors:** Alexander J. Millman, David W. Dowdy, Cecily R. Miller, Robert Brownell, John Z. Metcalfe, Adithya Cattamanchi, J. Lucian Davis

**Affiliations:** 1 Division of Pulmonary and Critical Care Medicine, San Francisco General Hospital, University of California San Francisco, San Francisco, California, United States of America; 2 Curry International Tuberculosis Center, San Francisco General Hospital, University of California San Francisco, San Francisco, California, United States of America; 3 Department of Medicine, San Francisco General Hospital, University of California San Francisco, San Francisco, California, United States of America; 4 Department of Biostatistics and Epidemiology, University of California San Francisco, San Francisco, California, United States of America; 5 PRIME Residency Program, University of California San Francisco, San Francisco, California, United States of America; 6 Bloomberg School of Public Health, Johns Hopkins University, Baltimore, Maryland, United States of America; The Australian National University, Australia

## Abstract

**Background:**

Respiratory isolation of inpatients during evaluation for TB is a slow and costly process in low-burden settings. Xpert MTB/RIF (Xpert) is a novel molecular test for tuberculosis (TB) that is faster and more sensitive but substantially more expensive than smear microscopy. No previous studies have examined the costs of molecular testing as a replacement for smear microscopy in this setting.

**Methods:**

We conducted an incremental cost–benefit analysis comparing the use of a single negative Xpert versus two negative sputum smears to release consecutive adult inpatients with presumed TB from respiratory isolation at an urban public hospital in the United States. We estimated all health-system costs and patient outcomes related to Xpert implementation, diagnostic evaluation, isolation, hospitalization, and treatment. We performed sensitivity and probabilistic uncertainty analyses to determine at what threshold the Xpert strategy would become cost-saving.

**Results:**

Among a hypothetical cohort of 234 individuals undergoing evaluation for presumed active TB annually, 6.4% had culture-positive TB. Compared to smear microscopy, Xpert reduced isolation bed utilization from an average of 2.7 to 1.4 days per patient, leading to a 48% reduction in total annual isolation bed usage from 632 to 328 bed-days. Xpert saved an average of $2,278 (95% uncertainty range $1582–4570) per admission, or $533,520 per year, compared with smear microscopy.

**Conclusions:**

Molecular testing for TB could provide substantial savings to hospitals in high-income countries by reducing respiratory isolation usage and overall length of stay.

## Introduction

Guidelines in high-income countries recommend initiating respiratory isolation of patients being evaluated for pulmonary tuberculosis (TB) pending the results of serial sputum smear microscopy. [1], [2] This multi-day process consumes a significant amount of patient time and hospital resources but has a limited yield, with only 4–10% of isolated inpatients generally found to have smear-positive TB. [3], [4], [5], [6], [7] An alternative to serial sputum examination is to use nucleic acid amplification tests (NAATs), which are more sensitive than microscopy and can be performed within hours. [8] The Centers for Disease Control and Prevention (CDC) has recommended that NAATs be incorporated routinely into TB diagnostic strategies including triaging inpatients out of respiratory isolation. [2] High-quality evidence shows that NAATs have high diagnostic accuracy [9] and rapid turn-around times [5], [10], and that they could have substantial clinical impact. [5], [10] Nevertheless, NAATs have not been widely adopted, particularly in the inpatient setting. One major concern about NAATs is that, until recently, no study had shown them to be cost-effective in high-income, low-incidence countries. [11], [12], [13], [14], [15], [16], [17], [18].

Xpert MTB/RIF (“Xpert”, Cepheid, Inc., Sunnyvale, CA) is a novel molecular diagnostic test with high sensitivity and specificity for pulmonary TB. [19] Xpert is simpler, faster, and less labor intensive than sputum smear microscopy and other commercial NAATs, and these characteristics have led to its increasing adoption in low- and middle-income countries where the burden of TB is high. [20], [21] Although Xpert is expensive, its high sensitivity and specificity for smear-positive TB on a single sputum sample make it attractive as a potential alternative to serial sputum smear microscopy to guide use of scarce and costly respiratory isolation rooms. The FDA recently approved the use of Xpert for detecting tuberculosis on July 25, 2013 [22]; however, there is limited information about its potential impact in various settings. Therefore, we conducted a cost-benefit analysis comparing Xpert to smear microscopy for guiding triage of inpatients being evaluated for TB at an urban public hospital in the U.S.

## Methods

### Patients

To inform development of the cost-benefit model, we reviewed medical records of consecutive patients undergoing evaluation for TB from January 1 through December 31, 2009, at San Francisco General Hospital, a university-affiliated urban public hospital. We defined any inpatient who underwent microbiologic testing for TB while in respiratory isolation as representative of the modeled population.

### Standard Diagnostic Strategy

Infection control policies at San Francisco General Hospital require that inpatients being evaluated for TB be placed in a negative-pressure respiratory isolation room until two sputum samples collected at least eight hours apart have been examined and found negative for acid-fast bacilli (AFB). A clinical laboratory scientist (CLS) in the central microbiology lab performs smear examination and reports the results once daily, seven days a week. Specimens received after 4 pm are processed the following day. Finally, in order to complete the microbiologic evaluation for TB, each patient should provide a third sputum sample for smear examination before or after discharge, and all three sputum samples should undergo mycobacterial culture and speciation. [23].

### Xpert Diagnostic Strategy

In comparison, we proposed an alternative strategy using Xpert testing of a single sputum sample to guide the triage decision. In this strategy, we considered a negative result on one sputum Xpert test to be equivalent to two negative sputum smear exams for the purpose of allowing discharge of a patient with possible TB from respiratory isolation, and, absent any ongoing indication for hospitalization, from the hospital. We assumed that any positive Xpert result would lead to continued respiratory isolation and initiation of anti-mycobacterial treatment, and that the time required for a clinician to discontinue respiratory isolation after a negative Xpert assay would be identical to the time required to discontinue respiratory isolation after a second negative smear examination. We further assumed that Xpert testing would be performed and reported once daily, seven days per week, on the same schedule as smear microscopy, and that three sputum samples would be obtained for mycobacterial culture and speciation to confirm the presence or absence of TB in all patients (after discharge if necessary). According to our model, patients assigned to the Xpert strategy would submit the sputum samples remaining after discharge directly to the laboratory, and would not require clinic visits for sputum collection. Thus, we assumed no difference in cost or health benefit between the Xpert and microscopy strategies with respect to the third smear examination and any of the mycobacterial cultures.

### Decision Analysis

**Figure 1:**
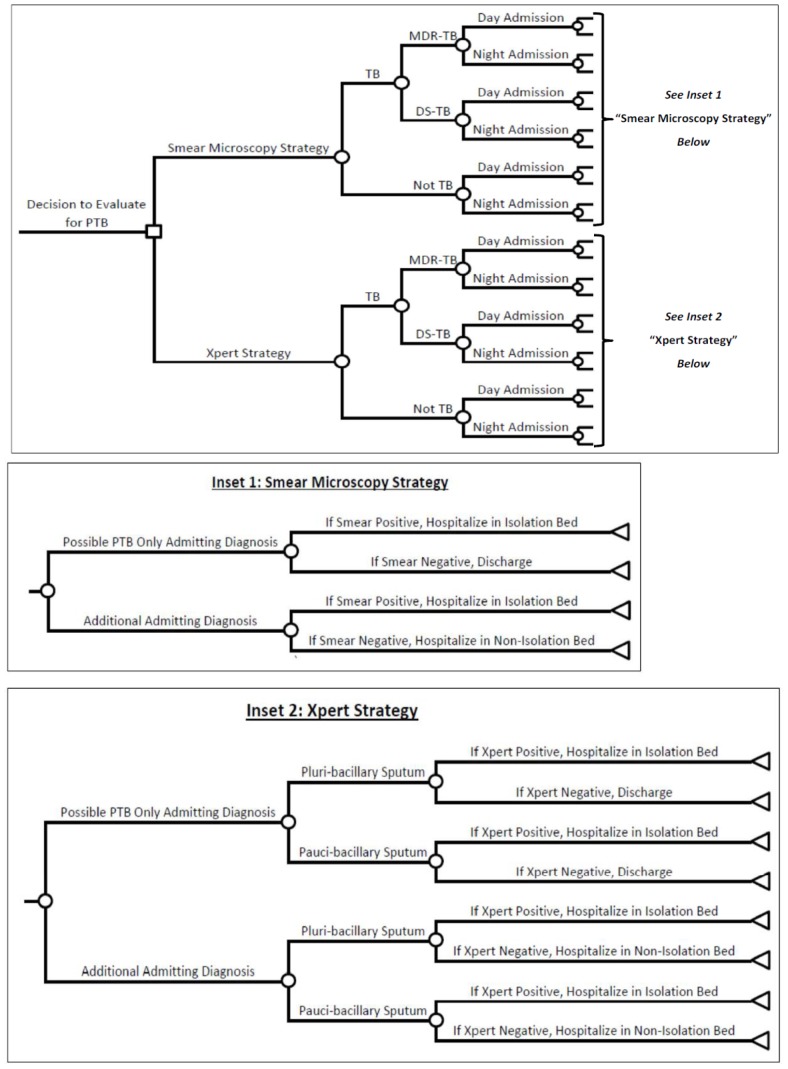
Decision analysis model comparing Xpert MTB/RIF and smear diagnostic strategies guiding respiratory isolation decisions. Definition of abbreviations: DS-TB = drug-sensitive TB. MDR-TB = multi-drug resistant TB. PTB = pulmonary TB. TB = tuberculosis. Legend: The large square represents a decision node, each circle a probabilistic node; and each triangle a terminal node. The two insets display the detailed distal branches leading to the terminal nodes for the two strategies being compared in the master tree. Applying the inputs in Tables 1 and 2, we used this decision analysis to generate the outcome data on economic and utilization costs presented in Tables 3 and 4. Note that for the Xpert strategy, the diagnostic performance of Xpert was estimated using the smear-positive sensitivity and specificity data for pluri-bacillary sputa and using the smear-negative sensitivity and specificity data for pauci-bacillary sputa. [19].

We developed a decision-analysis model comparing these two diagnostic strategies for guiding respiratory isolation decisions in inpatients being evaluated for active pulmonary TB (Figure 1). Our primary outcome was the incremental net monetary benefit of the Xpert strategy relative to the smear strategy. Since we expected incremental gains in health to be minimal in comparing these two approaches, we calculated incremental net monetary benefit as the net cost of the smear microscopy strategy minus the net cost of the Xpert strategy. We also considered savings in utilization costs as a secondary outcome, including reductions in (unnecessary) hospitalization and respiratory isolation. Within each strategy, we applied a cost penalty for false-positive screening results to reflect the unnecessary use of respiratory isolation, and for false-negative results to reflect the additional cost of contact investigation and treatment of transmitted TB (Online Supplement S1). We obtained base-case estimates of all epidemiologic and cost inputs (Table 1) through primary data collection and review of the literature (Online Supplement S1). We then aggregated the probabilities, utilization costs, and economic costs at each stage of evaluation and management to obtain unit individual and total annual estimates of cost, length of stay, and duration of respiratory isolation for each strategy. Finally, we calculated differences in economic and utilization costs between strategies. We adopted a health-system perspective, considering all economic costs of providing these health care services and the utilization costs of hospital beds and respiratory isolation rooms.

**Table 1:** Input variables for epidemiologic and diagnostic parameters used in the base-case and sensitivity analyses.

Variable	Base Value	Range	Reference
**Epidemiologic Parameters**			
TB prevalence among patients evaluated for TB (%)	6.4	0.5–15	Clinical data†
Proportion admitted during the daytime* (%)	24	10–50	Clinical data‡
No indication for admission except TB evaluation (%)	13	0–25	Clinical data‡
Number of annual inpatient TB evaluations	234	50–1000	Clinical data†
**Diagnostic Parameters**			
Sensitivity of Xpert (%)			
Smear-positive specimens	98	97–99	[19]
Smear-negative specimens	68	59–75	[19]
Specificity of Xpert (%)			
Smear-positive specimens	98	92–100	[19]
Smear-negative specimens	98	97–99	[19]
Sensitivity of smear microscopy (%)	78.5	65–92	[24]
Specificity of smear microscopy (%)	98	97–99	[24]

**Definition of abbreviations:** TB = tuberculosis.

**Legend:**

* Admitted between 9 am–4 pm.

† Microbiology laboratory database and patient chart review.

‡ SFGH inpatient admission database.

### Sensitivity and Uncertainty Analyses

To test the robustness of our model and identify the inputs that most contributed to costs and benefits, we performed one- and two-way sensitivity analyses for all parameters, using the ranges shown in Tables 1 and 2. We then performed probabilistic uncertainty analyses in which values from all parameters were simultaneously drawn from triangular distributions bounded by the highest and lowest plausible input values for each variable. We defined the 95% uncertainty range (UR) as the 2.5^th^ and 97.5^th^ percentiles of 10,000 such Monte Carlo simulations. We used TreeAge Pro Edition 2012 (TreeAge Software, Inc. Williamstown, MA) for all economic analyses.

**Table 2:** Input variables for cost parameters used in the base-case and sensitivity analyses.

Variable	BaseValue	Range	Reference
**Cost Parameters** *			
Xpert cost per test	$218	$10–$1,000	
Device (annualized over10 years)	$59	–	Cost data‡
Maintenance†	$64	–	Cost data‡
Cartridge	$60	–	Cost data‡
Labor	$35		
Cost per hour (salary andbenefits)	$60	–	Cost data§
Minutes per test	35	–	Observation
Smear cost per test	$10	$1–$100	
Materials	$2.50	–	[14], [25]
Labor	$7.50		
Cost per hour (salary andbenefits)	$60	–	Cost data‖
Minutes per test	7.5	–	Observation
Hospital bed cost per day	$2,292	500–5,000	Cost data‖
Marginal cost respiratoryisolation per day	$1,527	0–2,000	Cost data‖
Cost of four-drug anti-TBtherapy per day	$4.55	–	Cost data**

**Definition of abbreviations:** TB = tuberculosis.

**Legend:**

* In 2009 US Dollars.

† Includes full-service coverage after expiration of one-year warranty.

‡ Cepheid Schedule of Fees (July 2011).

§ State of California Employee Salary Database.

‖ San Francisco General Hospital (SFGH) Department of Finance Charge Database.

** SFGH Department of Pharmacy Drug Database.

### Human Subjects Considerations

The study was approved by the Committee on Human Research at the University of California, San Francisco. The Committee exempted the investigators from obtaining written informed consent on grounds that data collection for study inputs posed minimal risk to subjects.

## Results

### Patient Population

Between January and December, 2009, 234 patients were admitted and placed in respiratory isolation at San Francisco General Hospital for evaluation of pulmonary TB. TB evaluation was the sole reason for admission in 30 (13%) patients. The majority of admissions (76%) occurred during evening or nighttime lab hours (4 pm–9 am). Fifteen of the 234 (6.4%) patients were AFB smear-positive, and none had MDR-TB. If this cohort had hypothetically undergone Xpert testing, we estimated that these 15 and four additional patients without TB would have tested positive.

### Cost-benefit Analysis

Tables 1 and 2 show the key input variables for the decision analysis model, including base-case estimates and the ranges of values considered in one-way sensitivity analyses. After applying all costs of start-up, diagnostic evaluation, hospitalization, overhead, and treatment, the model predicted an average savings of $2,278 (95% UR $1582–4569) per inpatient admission for the Xpert strategy ($15,503 per admission) compared to the smear microscopy strategy ($17,783 per admission; Table 3) in the base-case scenario. As expected, these savings differed by TB status, with the Xpert strategy costing $205 more (95% UR $75–237) than the smear microscopy strategy among TB patients and $2,450 less (95% UR $964–2570) among non-TB patients. When we aggregated net costs for each strategy for all patients in the hypothetical cohort over an entire year, we projected substantial savings of $533,520 annually using the Xpert strategy compared to the smear strategy (Table 4).

**Table 3:** Average utilization and economic costs per patient.

Outcome	SmearStrategy	XpertStrategy	Difference	95% UncertaintyRange‡
**Length of Stay** *				
Isolation room	2.7	1.4	1.3	1.2, 1.3
Standard room	3.2	4.4	−1.2	−1.2, −1.1
Total	5.9	5.8	0.1	−0.1, 0.2
**Costs** †				
Isolation room	$10,483	$5,305	$5,178	$4,234, $5,200
Standard room	$7,285	$9,980	−$2,695	−$2,750, −$2,504
Diagnostictesting	$15	$218	−$203	−$699, −$66
Total	$17,783	$15,503	$2,278	$1,582, $4,570

**Legend:**

* In days;

† In 2009 US Dollars;

‡ 2.5–97.5% uncertainty range based on simultaneous sampling of all parameters of simultaneously drawn from triangular distributions bounded by the highest and lowest plausible input values for each variable in Tables 1 and 2.

Note that numbers displayed were subject to rounding.

**Table 4:** Total annual utilization and economic costs for all patients.

Outcome	SmearStrategy	XpertStrategy	Difference	95% UncertaintyRange‡
**Length of Stay** *				
Isolation room	632	328	304	281, 304
Standard room	749	1030	−281	−281, −257
Total	1,381	1,358	23	−23, 47
**Costs** †				
Isolation room	$2,453,022	$1,241,370	$1,211,652	$990,756, $1,216,800
Standard room	$1,704,690	$2,335,320	−$630,630	−$645,500, −$585,936
Diagnostictesting	$3,510	$51,012	−$47,502	−$163,655, −$15,444
Total	$4,161,222	$3,627,702	$533,520	$370,188, $1,069,380

**Legend:**

* In days;

† In 2009 US Dollars.

‡ 2.5–97.5% uncertainty range based on simultaneous sampling of all parameters of simultaneously drawn from triangular distributions bounded by the highest and lowest plausible input values for each variable in Tables 1 and 2.

Note that numbers displayed were subject to rounding.

The majority of the cost savings arose from reductions in length of stay in respiratory isolation. We projected that the Xpert strategy would on average reduce time spent in respiratory isolation by 1.3 days (95% UR 1.2–1.3 days; 1.4 isolation days with the Xpert strategy versus 2.7 days with the microscopy strategy), although average time in hospital would not necessarily decrease (5.8 hospital days with Xpert versus 5.9 with microscopy; Difference 0.1 days, 95% UR −0.1 to 0.2 days). Not surprisingly, projected length of stay also differed by TB status. TB patients were estimated to remain in hospital in respiratory isolation on average for 12.1 days after initiating treatment regardless of testing strategy, a length of stay driven by the severity of illness and by hospital infection-control guidelines for patients from congregate settings. Among non-TB patients, we projected an average stay in isolation of 0.7 days with the Xpert strategy and 2.1 days with the smear microscopy strategy, for a savings of 1.4 hospital days (95% UR 1.1–1.6) with the Xpert strategy. The average length of stay in hospital for non-TB patients did differ by evaluation strategy but to a lesser degree: 5.5 days with Xpert and 5.3 days with smear microscopy (Difference 0.2 days, 95% UR 0.2–0.3 days).

Based on these individual findings, our model also projected substantial reductions in total annual utilization of respiratory isolation beds among all patients (Table 4). Patients spent a total of 632 days in isolation when evaluated with the smear strategy versus an expected 328 days with Xpert (Difference 304 isolation days, 95% UR 281–304 days), a projected 48% decrease. Thus, as further shown in Tables 3 and 4, the number of days spent in respiratory isolation was the primary factor influencing average and total costs.

### Sensitivity Analyses

**Figure 2:**
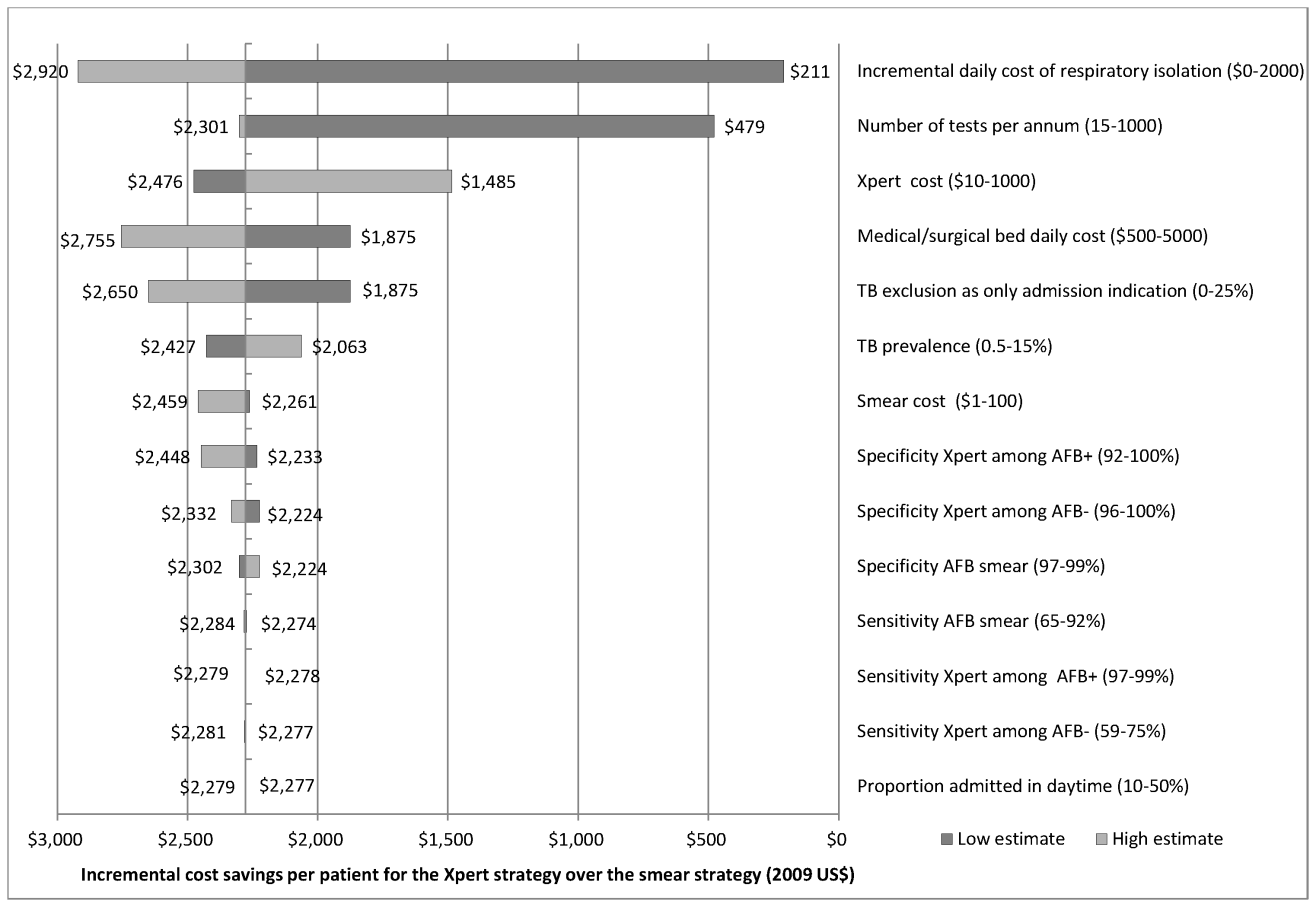
One-way sensitivity analyses comparing Xpert MTB/RIF and smear diagnostic strategies. Definition of abbreviations: AFB+, sputum acid-fast bacli smear-positive; AFB−, sputum acid-fast bacilli smear-negative. Legend: ^†^Ranges for sensitivity analyses are shown in parentheses. The vertical line at $2278 corresponds to the incremental savings in total costs per patient as shown in Table 3, dark grey bars show the estimated incremental savings of Xpert versus sputum smear when using the lowest value of the parameter range shown, and the light grey bars show the corresponding estimate when using the highest parameter value.

As shown in the one-way sensitivity analyses in Figure 2, the Xpert strategy was cost-saving relative to the serial smear strategy across a broad range of assumptions about the expenses of hospitalization, respiratory isolation, and diagnostic testing, including Xpert cost. In addition, our findings were robust to changes in Xpert sensitivity and specificity across the range of summary estimates reported in the literature, and to volume of tests performed, with the smear strategy favored only when the number of TB tests performed annually was less than 14. Finally, a two-way sensitivity analysis around the two variables found to be most influential – incremental daily cost of respiratory isolation and the number of tests performed per year – showed the Xpert strategy to be cost saving for most combinations of these two inputs. However, as the cost of isolation decreased, more tests needed to be performed annually for the Xpert strategy to achieve cost savings relative to the smear strategy (Figure S1). In a second two-way sensitivity analysis incorporating the cost of hospitalization and the incremental cost of an isolation room, we found that even if there were no additional cost for a respiratory isolation room, the cost of a regular acute hospital bed would have to decrease by over 50% - from $2292 to $1095 per day – to favor the smear microscopy strategy over the Xpert strategy.

## Discussion

Active pulmonary disease and other infectious forms of TB are infrequent among inpatients in the U.S. and other high-income countries, yet excluding these conditions consumes a substantial amount of patient time and hospital resources. Although molecular testing has the potential to speed such evaluations, concerns about the high costs of implementing these tests have prevented them from being used widely. [15], [16] This analysis suggests that the routine adoption of Xpert, a novel automated NAAT, to guide triage of inpatients undergoing evaluation for presumed pulmonary TB could reduce the cost of respiratory isolation by $2,278 per inpatient admission. This would save a medium-sized urban public hospital like ours approximately $533,520 per year.

This is the first study to find that using a NAAT for TB evaluation is cost-saving in a low-burden, high-income setting. Dowdy and colleagues examined how the GenProbe MTD test could be used as an add-on test for confirming *M. tuberculosis* complex in smear-positive specimens in a university hospital in the U.S., and found that the technical complexity of the manual GenProbe assay contributed to high labor costs and slow turn-around times, thereby blunting the potential benefit of their NAAT strategy. [13] Similarly, Rajalahti *et al* evaluated the cost-effectiveness of Cobas Amplicor PCR assay as an add-on test for evaluating isolation and treatment decisions for patients with presumptive TB in a Finnish hospital. [26] Although they found NAAT inexpensive in this scenario, they only performed NAATs twice weekly and on average waited four days for results; sensitivity analyses found that the strategy would have been cost-saving had testing been performed daily.

The key advantage of Xpert over prior NAATs is that its simplicity and automated nature enables real-time instead of batched testing, leading to more rapid availability of results. Indeed, we found that Xpert was cost-saving even though the costs of Xpert testing were similar to those reported for NAATs in previous studies [13], [14], [25], [26], because it reduced the number of respiratory isolation days. Because the incremental cost of respiratory isolation is high at our institution, Xpert’s ability to reduce respiratory isolation time by 1.4 days led to a 14% reduction in the overall costs of hospitalization compared with the standard smear strategy. In contrast, previous studies found that NAATs increased costs by 13–51% [13], [14], [26], primarily because of the high costs of testing and the low costs of isolation and hospitalization. Although the incremental cost of a respiratory isolation bed may vary widely between institutions, we found that even if respiratory isolation were free, the Xpert strategy would still be favored until the cost of a hospital bed day fell by over 50%. Although our sensitivity analyses show that the Xpert strategy is cost-saving across a range of inputs, the cost savings do depend on a minimal volume of testing at the hospital, primarily because the capital and maintenance costs of Xpert are spread across the total number of tests.

In addition to being cost-saving, Xpert has several other advantages over smear microscopy as a tool to guide triage of inpatients being evaluated for infectious TB. Xpert detects a large proportion of sputum smear-negative TB cases, and these contribute to disease transmission if undetected. [27] This is of particular concern in the hospital setting where exposure to TB may adversely affect a large number of health care workers and susceptible patients. Xpert is also highly specific for TB, which is potentially advantageous in low TB-burden settings where non-tuberculous mycobacteria may be more common. Finally, the use of Xpert also provides significant unmeasured individual benefits to patients in low prevalence settings by allowing earlier discharge from respiratory isolation and potentially earlier discharge from the hospital. These benefits include increased contact with staff while in the hospital, potentially decreased risk of healthcare-associated complications while hospitalized because of decreased time in the hospital, and faster return to work and family. Although the explicit inclusion of patient costs is beyond the scope of this analysis, we would expect shorter hospitalizations to directly reduce costs to uninsured, self-paying patients, while a faster return to work would reduce lost earnings from absenteeism. In low-burden settings such as ours, the vast majority of patients do not have TB and therefore stand to benefit from earlier exclusion of TB.

Our analysis has several limitations. We collected much of our input data from a single institution, which may limit the generalizability of our model, although sensitivity analyses showed the Xpert strategy to be cost-saving across a range of plausible inputs. Another limitation is that our institution requires only two negative sputa to discontinue respiratory isolation, whereas many other hospitals may require three negative sputa. However, Xpert would likely result in further cost savings for those hospitals because it would be able to reduce respiratory isolation time even more than predicted in our model. Finally, our model does not account for the fact that the same Xpert platform used for TB testing can also be used (with different modular cartridges) for U.S. Food and Drug Administration (FDA) - approved testing of other infections including influenza, *C. difficile* colitis, and methicillin-resistant *Staphylococcus aureus* (MRSA). In institutions that already utilize this platform, the incremental per-test cost of machine procurement and maintenance for TB evaluation could drop substantially.

In conclusion, we used a decision analysis model, coupled with primary data on costs and outcomes, to demonstrate that using Xpert to guide respiratory isolation decisions for patients undergoing evaluation for TB in an urban public hospital is likely to reduce health care costs and improve the patient experience by shortening the amount of time spent in respiratory isolation. The World Health Organization recently endorsed the use of Xpert for incremental case finding in low- and middle-income countries where TB incidence is high [20]; our analysis suggests that Xpert could also confer large benefits in low TB-incidence, high-income settings for the qualitatively different purpose of excluding TB. Given the robustness of our results to very wide parameter variations, implementation of Xpert is likely to reduce costs associated with respiratory isolation of patients being evaluated for TB at most hospitals in low-burden settings. Now that Xpert is FDA-approved for TB testing in the US, implementation data on actual costs and impact can further inform clinical and public health practice and future hospital infection-control guidelines on appropriate TB evaluation strategies.

## Supporting Information

Figure S1 **Two-way sensitivity analysis of the incremental cost per day of respiratory isolation and the number of TB tests per year. Legend:** The two-way sensitivity analysis on the incremental cost per day of respiratory isolation and the number of TB test per year. The area in blue is cost saving for the Xpert strategy and the area in pink is cost saving for the smear strategy for the estimates applied in the sensitivity analysis.
(TIFF)

Online Supplement S1 (DOC)
