# Supplementary figures and images for: Rapid Molecular Testing for TB to Guide Respiratory Isolation in the U.S.: A Cost-Benefit Analysis
Source: PLoS One. 2013 Nov 20;8(11):e79669. doi: 10.1371/journal.pone.0079669 (PMC3835836; doi:10.1371/journal.pone.0079669)

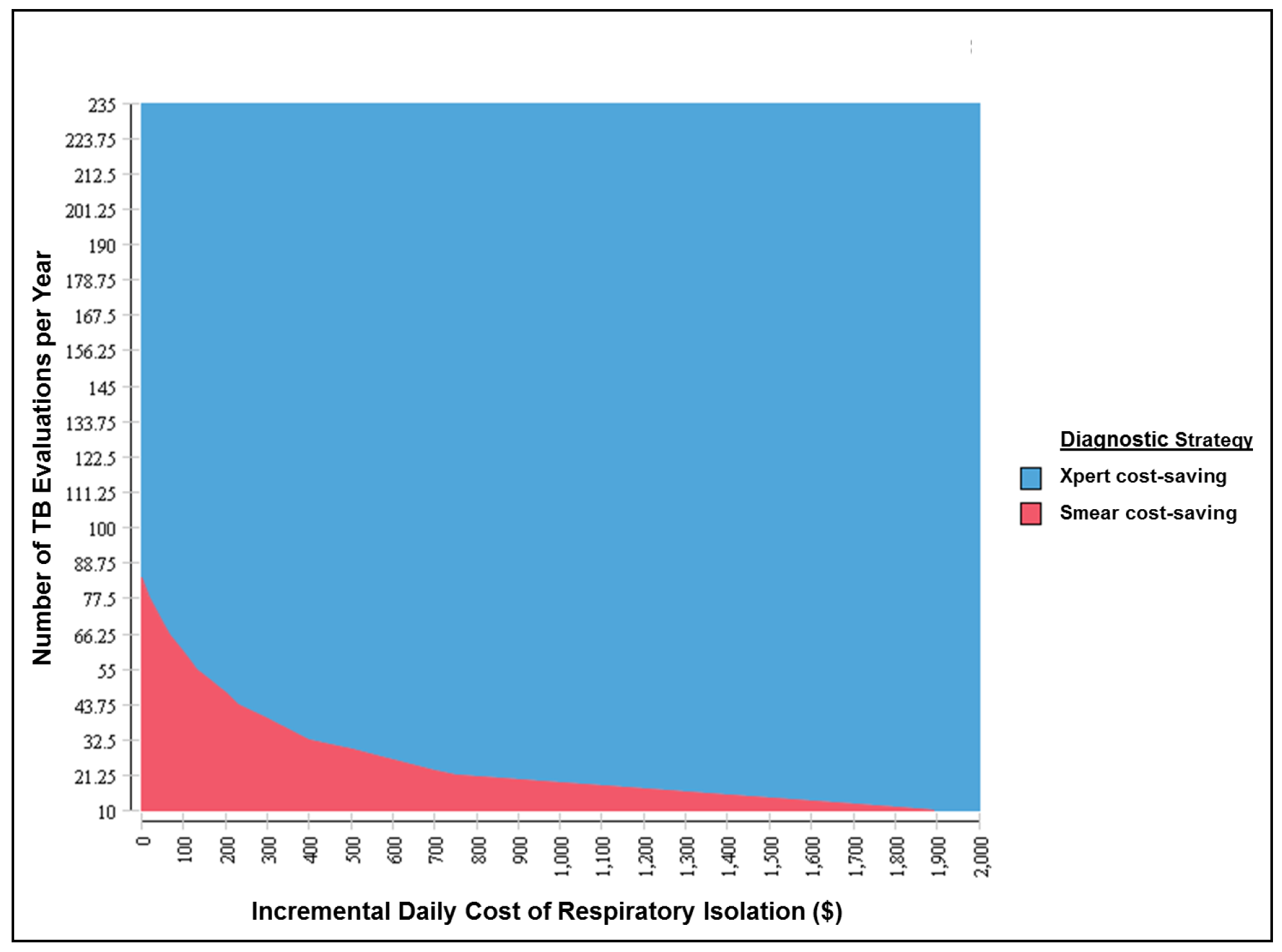

Supplement: Figure S1 — Two-way sensitivity analysis of the incremental cost per day of respiratory isolation and the number of TB tests per year. Legend: The two-way sensitivity analysis on the incremental cost per day of respiratory isolation and the number of TB test per year. The area in blue is cost saving for the Xpert strategy and the area in pink is cost saving for the smear strategy for the estimates applied in the sensitivity analysis. (TIFF) [file pone.0079669.s001.tif]
