# Supplementary material for: Rapid Molecular Testing for TB to Guide Respiratory Isolation in the U.S.: A Cost-Benefit Analysis
Source: PLoS One. 2013 Nov 20;8(11):e79669. doi: 10.1371/journal.pone.0079669 (PMC3835836; doi:10.1371/journal.pone.0079669)
Supplement: Online Supplement S1 — (DOC) [file pone.0079669.s002.doc]

**Supporting Information**

**Methods**

*Inputs to the decision-analysis model*

We obtained sensitivity and specificity estimates for Xpert for smear-positive and smear-negative patients from a published Cochrane systematic review and meta-analysis which included 18 studies. [S1] We estimated the sensitivity and specificity of sputum smear microscopy based on a published systematic review of the literature [S2], and confirmed these estimates by calculating the sensitivity and specificity of sputum smears done on all inpatients at San Francisco General Hospital in the clinical microbiology laboratory for the diagnosis of culture-positive TB in 2009.

We linked hospital databases maintained by the admitting office, the microbiology laboratory, and facilities management to identify all inpatients admitted to respiratory isolation rooms as part of an evaluation for pulmonary TB in 2009. We estimated each individual's overall length of stay in hospital and in respiratory isolation.

We obtained base-case estimates of the number of inpatients isolated and evaluated for infectious TB, and of the number of active pulmonary TB cases, by reviewing the patient databases of the hospital’s clinical microbiology laboratory for the year 2009, and cross-referencing patient locations for these episodes with a facilities-management list of all negative-pressure respiratory isolation rooms. We cross-referenced the daily inpatient census database to determine admission times and estimate length of stay in the hospital and in respiratory isolation. We extracted hospital admission times from databases maintained by the hospital. We reviewed medical records for all patients admitted to respiratory isolation to determine if exclusion of infectious TB was the primary reason for admission (as it was for some patients residing in congregate settings) or whether there were additional medical indications for admission. We estimated the length of stay for each admission diagnosis by searching a hospital administrative database using ICD-9 codes and the terms “infectious tuberculosis,” “tuberculosis,” “pulmonary tuberculosis,” “pneumonia,” “atypical respiratory infection,” and “hemoptysis.” We added an extra 0.5 days to the length of stay of subjects admitted after the daily 4pm deadline for processing sputum in the lab, assuming that these specimens would be submitted at randomly-distributed times over the following 24 hours.

*Cost estimates*

We estimated the incremental cost of implementing the proposed Xpert testing program in our hospital’s clinical microbiology lab, using the inputs in Table 2. We obtained the costs of purchasing the Xpert platform (includes the machine, printer, and power supply), an annual maintenance contract, and cartridges directly from the manufacturer, according to the manufacturer’s standard schedule of fees in July 2011. We assumed a 10-year useful life for the Xpert platform and factored in a 3% annual depreciation. These costs were adjusted to 2009 USD and used to calculate the cost per test [S3]. As required by the Clinical Laboratory Improvement Association (CLIA) based on Xpert’s classification as a moderate-complexity assay, we budgeted for a clinical laboratory scientist (CLS) to perform testing in the Xpert arm, as well as in the smear microscopy arm, as is standardly done. We estimated the annual cost of a CLS using publicly available records of employee salaries from the State of California. We estimated assay turn-around times by directly observing hospital staff in the microbiology laboratory preparing and examining sputum smears, and running the *Clostridium difficile* Xpert assay, a similar procedure to Xpert testing for TB.

The cost of a hospital bed-day was unavailable, so we estimated it by multiplying the daily charge in 2009 USD provided by the hospital’s financial office by the institutional cost-to-charge ratio of 0.43 reported to state regulatory authorities in 2009. [S4]. We assumed that this amount encompassed the cost of facilities, supplies, equipment for respiratory isolation, and staffing (physician fees and nurse and ancillary staff salaries) during a 24-hour period. We obtained the daily cost of TB medications from the hospital pharmacy.

We applied a cost penalty of $940 to false-negative Xpert results to account for additional inpatient TB transmission and TB screening of exposed patients and staff associated with an earlier and/or incorrect discharge from respiratory isolation as compared with the smear strategy. We handled false-positive Xpert results in the same manner as false-positive smear microscopy results. Patients with an Xpert result screening positive for MDR-TB would have confirmatory drug-susceptibility testing performed on the culture and would be treated with an appropriate drug regimen.

**References for Supporting Information**

S1. Steingart KR, Sohn H, Schiller I, Kloda LA, Boehme CC, et al (2013) Xpert(R) MTB/RIF assay for pulmonary tuberculosis and rifampicin resistance in adults. Cochrane Database Syst Rev 1: CD009593.

S2. Steingart KR, Henry M, Ng V, Hopewell PC, Ramsay A, et al (2006) Fluorescence versus conventional sputum smear microscopy for tuberculosis: A systematic review. Lancet Infect Dis 6: 570-581.

S3. United State Department of Labor Consumer Price Index, Medical Care Component. Available at <http://www.bls.gov/cpi/>. Accessed 10/1/2011.

S4. State of California Office of Statewide Health Planning and Development Annual Financial Disclosure Reports. 2009 Hospital Annual Financial Disclosure Report. Available at: <http://siera.oshpd.ca.gov/annualfinancial.aspx>. Accessed 10/1/2011.
